# Pregnancy outcome in women before and after cervical conisation: population based cohort study

**DOI:** 10.1136/bmj.a1343

**Published:** 2008-09-18

**Authors:** Susanne Albrechtsen, Svein Rasmussen, Steinar Thoresen, Lorentz M Irgens, Ole Erik Iversen

**Affiliations:** 1 Department of Obstetrics and Gynecology, Haukeland University Hospital, Institute of Clinical Medicine, University of Bergen, 5021 Bergen, Norway; 2 Medical Birth Registry of Norway, Locus of Registry Based Epidemiology, Department of Public Health and Primary Health Care, University of Bergen and the Norwegian Institute of Public Health, Norway; 3 Institute of Clinical Medicine, University of Bergen, Bergen; 4 Cancer Registry of Norway, Oslo, Section for Pathology, Gade Institute, University of Bergen, Bergen

## Abstract

**Objectives** To examine the consequences of cervical conisation in terms of adverse outcome in subsequent pregnancies.

**Design** Population based cohort study.

**Data sources** Data on cervical conisation derived from the Cancer Registry of Norway and on pregnancy outcome from the Medical Birth Registry of Norway, 1967-2003. 15 108 births occurred in women who had previously undergone cervical conisation and 57 136 who subsequently underwent cervical conisation. In the same period there were 2 164 006 births to women who had not undergone relevant treatment (control).

**Results** The proportion of preterm delivery was 17.2% in women who gave birth after cervical conisation versus 6.7% in women who gave birth before cervical conisation and 6.2% in women who had not undergone cervical conisation. The relative risk of a late abortion (<24 weeks’ gestation) was 4.0 (95% confidence interval 3.3 to 4.8) in women who gave birth after cervical conisation compared with no cervical conisation. The relative risk of delivery was 4.4 (3.8 to 5.0) at 24-27 weeks, 3.4 (3.1 to 3.7) at 28-32 weeks, and 2.5 (2.4 to 2.6) at 33-36 weeks. The relative risk of preterm delivery declined during the study period and especially of delivery before 28 weeks’ gestation.

**Conclusion** Cervical conisation influences outcome in subsequent pregnancies in terms of an increased risk of preterm delivery, especially in the early gestational age groups in which the clinical significance is highest. A careful clinical approach should be taken in the selection of women for cervical conisation and in the clinical care of pregnancies after a cervical conisation.

## Introduction

Many countries have widespread screening programmes entailing cytological examination of the cervix. Only a minority of cervical intraepithelial neoplasia lesions will eventually develop into invasive cancers^1^ but, in the absence of precise prognostic factors, women usually undergo cervical conisation when the diagnosis is confirmed. Although both age at start of screening and the interval between screenings differ between countries, most countries have observed a measurable impact on the incidence cervical cancer with a standardised systematic approach.

As more women are treated and as maternal age is increasing during recent years, the likelihood of having a cervical conisation in the active reproductive period is also increasing. Concern has been raised about the consequences of conisation in terms of adverse pregnancy outcome. With techniques such as laser conisation and large loop methods complications in pregnancy have been reported as less common.^2^

Most studies on adverse pregnancy outcome after cervical conisation were designed as case-control studies or were small, comprising a low number of cases, and randomised trials have not been performed. A recent meta-analysis by Kyrgiou et al showed a significantly increased risk of preterm delivery, low birth weight, and premature rupture of membranes.^2^ Even in this meta-analysis, the conclusions were based mostly on small numbers in the subgroups. With limited information on the effect of confounding factors, the question remains whether adverse outcomes are related to characteristics of women rather than to the treatment itself.

In Norway, we linked data from the medical birth registry and the cancer registry to perform a national registry based cohort study with a large sample size. We assessed effects of cervical conisation on gestational age at delivery and birth weight. We also clarified whether the effects were related to the cervical conisation itself or to other factors. During the observation period methods of treatment changed and we wanted to assess secular trends.

## Methods

We linked data from the two registries by the national identification number.

### Exposure

Since 1953, the cancer registry has collected information on all cancer diagnoses as well as premalignant lesions, including intraepithelial neoplasia with staging. The compulsory reporting system is based on clinical, pathology, and cytology reports. During 1953-79 and from 1986 onwards, treatment of intraepithelial neoplasia with cervical conisation has also been notified, though without specification of surgical method. During 1980-5, only data on histological diagnoses—that is, the grade of intraepithelial neoplasia—were notified and we excluded these women from the exposed group and included them in the not treated group. The method used—knife, laser, or large loop conisation—could not be identified in the individual woman. Until 1980, all treatment was knife conisation. Since 1985, laser based methods have been used to an increasing extent, and loop electrosurgical excision of the cervix was introduced in 1990-5. We included in the exposed group all women aged less than 45 at the time of cervical conisation.

### Outcome

Established in 1967, the birth registry comprises compulsory notification of all live births and stillbirths in Norway from 16 completed weeks of gestation.^3^ A standardised notification form is used, including demographic variables and data on maternal health, reproductive history, complications during pregnancy and delivery, and neonatal outcome. The notification form, filled in by the midwife or physician attending the delivery, is sent to the registry within nine days after birth or at discharge from the delivery or neonatal care unit.

Calculation of gestational age was based on the first day of the last menstrual period. Until 1998, gestational age based on ultrasonography was not recorded. During 1998-2003, gestational age was based on ultrasonography when the date of the last menstrual period was missing. We removed outliers in gestational age using a linear regression approach in which gestational age was regressed against birth weight in strata of whole weeks of gestation. This did not significantly change the results and uncorrected observations were used consistently. The proportion of women with missing data on gestational age amounted to 5.3%, while data on birth weight were almost complete.

All fetuses delivered at <24 weeks’ gestation or with birth weight <500 g were classed as late abortion. Fetuses delivered at 24-36 weeks’ gestation or with birth weight 500-2499 g were classed as preterm delivery.

We categorised women with a cervical conisation according to whether they had been treated before or after the delivery; most (99.7%) were treated before start of the index pregnancy. To control for confounding factors that otherwise could be difficult to account for, we followed two reference cohorts, in addition to the exposed cohort, with respect to preterm birth: women who had never had cervical conisation (non-exposed) and women who underwent cervical conisation after delivery.

The present study included births from 1967 to 2003. Table 1 gives the numbers of exposed and non-exposed women.[Table tbl1]

**Table 1 tbl1:** Birth related characteristics of births in women with cervical conisation after and before delivery and births in women with no cervical conisation, Norway 1967-2003. Figures are numbers (percentages) of births

Birth related characteristics	Births after cervical conisation	Births before cervical conisation	No cervical conisation
Year of birth:			
1967-79	557 (3.7)	24 194 (42.3)	767 013 (35.4)
1980-9	1371 (9.1)	19 231 (33.7)	511 398 (23.6)
1990-9	7718 (51.1)	13 076 (22.9)	585 144 (27.0)
2000-3	5462 (36.2)	635 (1.1)	300 451 (13.9)
Total	15 108 (100)	57 136 (100)	2 164 006 (100)
Maternal age at cervical conisation (years):			
<25	4759 (31.5)	3526 (6.2)	—
25-34	9807 (64.9)	28 071 (49.1)	—
>34	542 (3.6)	25 539 (44.7)	—
Total	15 108 (100)	57 136 (100)	—
Maternal age at delivery (years):			
<25	679 (4.5)	28 310 (49.5)	721 152 (33.3)
25-34	10 877 (72.0)	26 442 (46.3)	1 231 109 (56.9)
>34	3552 (23.5)	2384 (4.2)	211 645 (9.8)
Total	15 108 (100)	57 136 (100)	2 163 906* (100)
Birth order:			
1	4207 (27.8)	26 259 (46.0)	891 989 (41.2)
2	5695 (37.7)	19 862 (34.8)	748 375 (34.6)
≥3	5206 (34.5)	11 015 (19.3)	523 642 (24.2)
Total	15 108 (100)	57 136 (100)	2 164 006 (100)

*Includes 100 cases for which we had no information on maternal age.

The national identification number allowed linkage with the Central Population Registry and the Cause of Death Registry, ensuring complete ascertainment of all births as well as perinatal deaths.

### Statistics

We used relative risk to estimate associations of preterm birth with cervical conisation and adjusted odds ratios, obtained from logistic regression, to calculate approximate adjusted relative risks.^4^ The population attributable risk percentage (PAR%) was calculated and refers to the percentage of cases attributable to the cervical conisation.^5^

Confidence intervals for proportions were calculated by the score method.^6^ We compared z scores of birth weight in women with a conisation before and after pregnancy or not. Z scores were calculated by regression of power transformed birth weight against gestational age using fractional polynomials; adding sex and birth order (1 or 2+) to the model.^7^ The method of scaled absolute residuals was used to model standard deviation (SD) used in the calculations of z scores against gestational age.^7^

The data linkage between the birth registry and the cancer registry was notified to the Norwegian Data Inspectorate. We used the statistical package for the social sciences, version 13.0 (SPSS, Chicago, IL).

## Results

From 1967 to 2003, 0.7% of the births in the population studied occurred in women who had undergone a cervical conisation before the index pregnancy and 2.6% after. Births after a cervical conisation were more common in older women and with higher birth orders (table 1).[Table tbl1] The proportion of preterm birth (delivery before 37 weeks’ gestation) was 17.2% (95% confidence interval 16.6% to 17.8%) in women who gave birth after cervical conisation, 6.7% (6.5% to 6.9%) in women gave birth before cervical conisation, and 6.2% (6.2% to 6.3%) in women who did not have conisation.

The relative risk of premature delivery in women after a cervical conisation compared with women who did not have cervical conisation increased with decreasing gestational age (table 2). Also, the risk of late abortion was higher after a cervical conisation. The relative risk decreased slightly after adjustment for maternal age and birth order (table 2).[Table tbl2] The same pattern was observed according to birth weight (data not shown).

**Table 2 tbl2:** Numbers and proportions of preterm deliveries with relative risks (95% confidence intervals) in births of women with cervical conisation and no cervical conisation by gestational age in Norway, 1967-2003

Gestational age (weeks)	Births after cervical conisation	Births before cervical conisation	No cervical conisation	Births after *v* births before cervical conisation				Births after cervical conisation *v* no cervical conisation	
				RR (95% CI)	Adjusted*	Adjusted†		RR (95% CI)	Adjusted*
Late abortion	226 (1.5)	209 (0.4)	8501 (0.4)	4.0 (3.3 to 4.8)	3.2 (2.6 to 3.8)	3.2 (2.6 to 3.9)		4.0 (3.3 to 4.8)	3.3 (2.9 to 3.7)
24-27	234 (1.5)	263 (0.5)	7757 (0.4)	3.3 (2.8 to 4.0)	3.3 (2.7 to 3.9)	3.0 (2.5 to 3.7)		4.4 (3.8 to 5.0)	4.3 (3.8 to 4.9)
28-32	535 (3.5)	614 (1.1)	22 945 (1.1)	3.3 (3.0 to 3.7)	3.2 (2.9 to 3.6)	3.0 (2.6 to 3.4)		3.4 (3.1 to 3.7)	3.4 (3.1 to 3.7)
33-36	1599 (10.6)	2724 (4.8)	95 764 (4.4)	2.3 (2.2 to 2.4)	2.2 (2.0 to 2.3)	2.2 (2.0 to 2.3)		2.5 (2.4 to 2.6)	2.4 (2.3 to 2.5)

*Adjusted for birth order (1 *v* >1) and maternal age at delivery.

†Adjusted for birth order (1 *v* >1) and maternal age at treatment.

Births in women without cervical conisation and with conisation after delivery had similar distribution according to gestational age, whereas delivery at lower gestational ages was more common in women with cervical conisation (fig 1). The distribution according to birth weight showed a different pattern (fig 2). Birth weight in women who gave birth after conisation was lower than in those who had not had conisation. The relative risk of a preterm birth, however, was lower when compared with women with a conisation after delivery, particularly the relative risk of delivery at 24-27 weeks, which was reduced from 4.3 to 3.0 (table 2).[Table tbl2]

**Figure fig1:**
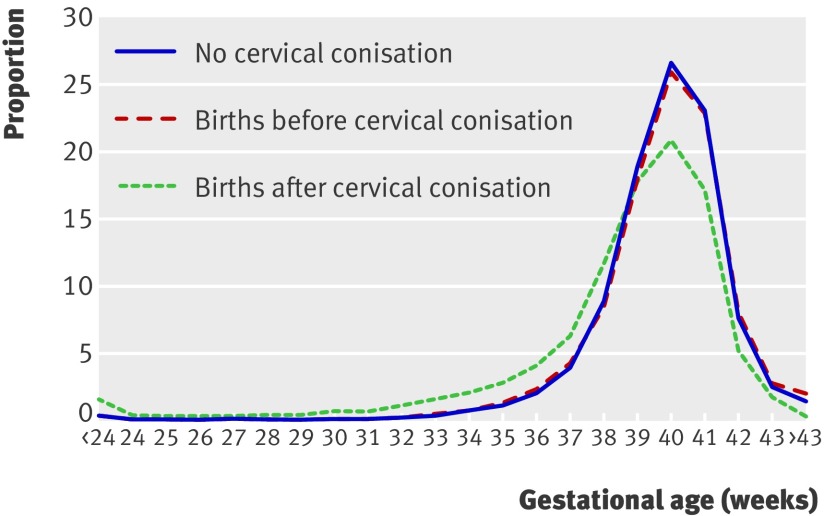
**Fig 1** Births before and after cervical conisation or with no cervical conisation by gestational age, Norway 1967-2003

**Figure fig2:**
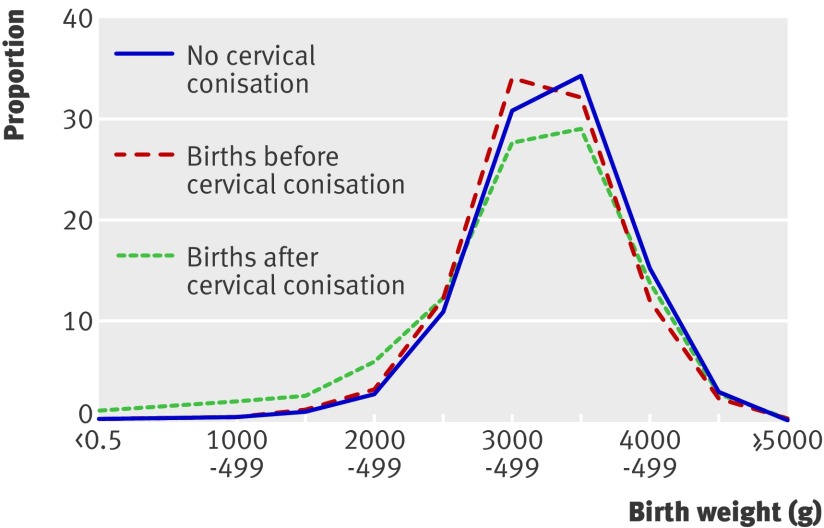
**Fig 2** Births before and after cervical conisation or with no cervical conisation by birth weight, Norway 1967-2003

Infants born to women who had a conisation after delivery were lighter than those born to women without a conisation. In women with no cervical conisation, z scores were on average 0.004 (95% confidence interval 0.002 to 0.005) compared with −0.04 (−0.058 to −0.023) in births after a conisation (data not presented). The lowest z score −0.135 (−0.144 to −0.127) was found in births before a conisation.

During the study period, the excess risk of a preterm delivery in women who underwent cervical conisation decreased, particularly the risk of delivery before 28 weeks (fig 3).

**Figure fig3:**
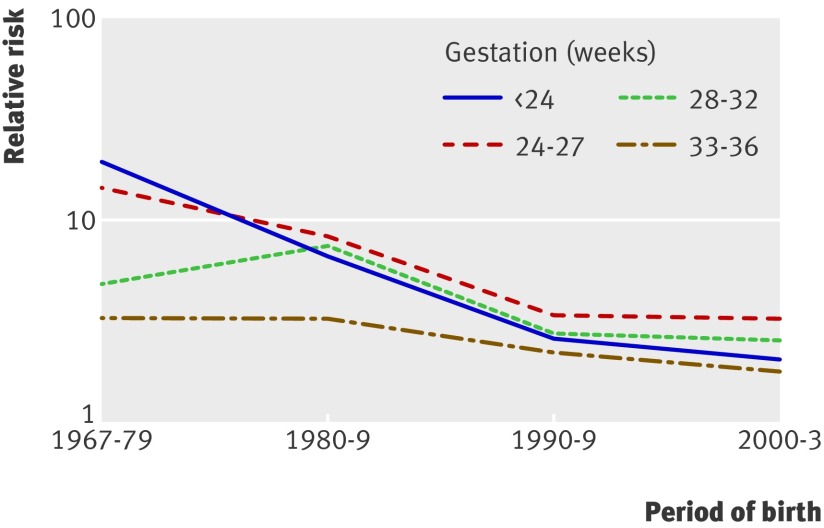
**Fig 3** Relative risk of preterm birth in various gestational age groups in women who gave birth after cervical conisation compared with births to women with no cervical conisation by year of birth, Norway 1967-2003

In women aged under 25 at the time of treatment, preterm delivery was no more common than in older women (table 3).

**Table 3 tbl3:** Preterm delivery (<37 weeks) by maternal age at delivery and at treatment with cervical conisation, Norway 1967-2003. Figures are numbers (percentages) of births

	Births after cervical conisation	Births before cervical conisation	No cervical conisation
Maternal age at delivery (years):			
<25	134 (19.7)	1908 (6.7)	44 999 (6.2)
25-34	1857 (17.1)	1715 (6.5)	72 919 (5.9)
≥35	603 (17.0)	187 (7.8)	17 026 (8.0)
Total	2594 (17.2)	3810 (6.7)	134 944* (6.2)
Maternal age at treatment (years):			
<25	842 (17.7)	295 (8.4)	—
25-34	1667 (17.0)	1883 (6.7)	—
≥35	85 (15.7)	1632 (6.4)	—
Total	2594 (17.2)	3810 (6.7)	—

*Includes 23 cases for which we had no information on maternal age.

The population attributable risk percentage of preterm delivery attributable to cervical conisation before 28, 33, and 37 weeks of gestation was 2.0%, 1.7%, and 1.2%, respectively.

## Discussion

In this cohort study, based on 15 108 births to women who had undergone cervical conisation, we found an increased risk of preterm delivery after a cervical conisation because of intraepithelial neoplasia. The excess risk was highest for late abortion and for preterm delivery before 33 weeks, in agreement with a cohort study from Finland.^8^ The high risk early in pregnancy is clinically significant. In previous studies on pregnancy outcome after a cervical conisation, the small numbers of cases have hampered the ability to detect significant differences between gestational age groups.^9^ ^10^ ^11^

The population attributable risk percentage of preterm birth because of cervical conisation was not high. Women who have had cervical conisation can easily be identified in a clinical setting, however, and might benefit from closer surveillance during pregnancy to improve the outcome. Focus on such women seems to be a sensible strategy for reducing the total number of preterm deliveries. Also, optimised surgical treatment of the cervix to avoid or reduce cervical damage might be beneficial.

### Strengths and weaknesses

Information bias was low in our cohort study, which included all births in Norway; neither data on exposure nor on outcome were derived directly from the women but from clinical sources. The exposure, cervical conisation, was clearly defined. Misclassification of exposure—for instance, omission of notification—would, to the extent it might occur, not influence the relative risks. Complete follow-up of all exposed women represents another strength.

The excess risk of preterm delivery, however, could be caused by factors other than the cervical conisation itself—factors that might characterise the exposed group. To avoid such confounding, we used a group of women who had cervical conisation after delivery as a reference, in addition to all women who had never had conisation. Although the two reference cohorts might have a different distribution of possible confounders, there was virtually no difference between the two groups with respect to gestational age. These two reference cohorts enabled us to control for confounding factors that otherwise could be difficult to account for.

Smoking is a potential confounding variable, and relevant data were not available in the registries. The difference in birth weight in the three groups of women could partly be explained by different smoking habits. Smoking during pregnancy increases the occurrence of low birth weight. Women who smoke also have a higher risk of developing intraepithelial dysplasia and thus are more likely to have a cervical conisation. In the present study, births in women who later underwent cervical conisation virtually had the same distribution of gestational age as births in women who never had cervical conisation, though with birth weight shifted to the left, consistent with different smoking habits. Several studies have used birth weight as an outcome variable.^2^ ^10^ ^12^ ^13^ Our results indicate that the effect of cervical conisation could be overestimated if birth weight is used as an outcome variable, possibly because of confounding by different smoking habits.

The time trend described could be explained by the fact that over the period studied, smaller amounts of cervical tissue were removed as new methods of conisation were introduced. Thus, the increased risk of preterm birth might be related to the mechanism by which cervical tissue is removed. The time trend was not explained by a trend in the general population towards fewer preterm births as the opposite has been observed.^14^

In the study period, the mean maternal age at delivery increased in all birth orders and women had fewer births.^14^ The influence of birth order and maternal age on the risk of preterm birth was rather limited. On the other hand, because of the increasing mean maternal age at delivery, a higher number of pregnant women would have had a previous cervical conisation.

The study underscores the need for a careful clinical approach to women with a previous cervical conisation when they become pregnant. Women, especially those who have not yet had children, should be informed about the increased risk of adverse pregnancy outcome in terms of increased occurrence of late abortion and preterm birth. This information should be kept in mind when counselling young women with a low grade cervical neoplasia and might support watchful waiting in this group of women, especially the youngest.

**What is already known on this topic**

Evidence from smaller studies suggests a significant increased risk of preterm delivery and low birth weight after cervical conisation

**What this study adds**

Cervical conisation increases the risk of preterm delivery, especially in the early gestational age groups, in which the clinical significance is highest

## Notes

Ethical approval: Not required (research ethics committees in Norway regularly exempt research on anonymised registry data from ethical review).

Provenance and peer review: Not commissioned; externally peer reviewed.

## Notes

**Cite this as:** *BMJ* 2008;337:a1343
